# Maternal selenium deficiency in mice promotes sex‐specific changes to urine flow and renal expression of mitochondrial proteins in adult offspring

**DOI:** 10.14814/phy2.14785

**Published:** 2021-03-26

**Authors:** Elliott S. Neal, Pierre Hofstee, Montana R. Askew, Nykola L. Kent, Lucy A. Bartho, Anthony V. Perkins, James S. M. Cuffe

**Affiliations:** ^1^ School of Biomedical Sciences The University of Queensland St Lucia QLD Australia; ^2^ School of Medical Science Griffith University Gold Coast Campus Southport QLD Australia

**Keywords:** DOHaD, fetal programing, maternal diet, offspring kidney, selenium

## Abstract

Selenium deficiency during pregnancy can impair fetal development and predispose offspring to thyroid dysfunction. Given that key selenoproteins are highly expressed in the kidney and that poor thyroid health can lead to kidney disease, it is likely that kidney function may be impaired in offspring of selenium‐deficient mothers. This study utilized a mouse model of maternal selenium deficiency to investigate kidney protein glycation, mitochondrial adaptations, and urinary excretion in offspring. Female C57BL/6 mice were fed control (>190 µg selenium/kg) or low selenium (<50 µg selenium/kg) diets four weeks prior to mating, throughout gestation, and lactation. At postnatal day (PN) 170, offspring were placed in metabolic cages for 24 hr prior to tissue collection at PN180. Maternal selenium deficiency did not impact selenoprotein antioxidant activity, but increased advanced glycation end products in female kidneys. Male offspring had reduced renal Complex II and Complex IV protein levels and lower 24 hr urine flow. Although renal aquaporin 2 (*Aqp2*) and arginine vasopressin receptor 2 (*Avpr2*) mRNA were not altered by maternal selenium deficiency, a correlation between urine flow and plasma free T_4_ concentrations in male but not female offspring suggests that programed thyroid dysfunction may be mediating impaired urine flow. This study demonstrates that maternal selenium deficiency can lead to long‐term deficits in kidney parameters that may be secondary to impaired thyroid dysfunction. Considering the significant burden of renal dysfunction as a comorbidity to metabolic diseases, improving maternal selenium intake in pregnancy may be one simple measure to prevent lifelong disease.

## Graphical abstract

Male offspring of selenium deficient mothers display reduced 24 hr urine flow, which is not driven by reduced 24 hr water intake or altered expression of *Aqp2* or *Avpr2* mRNA within the kidney.

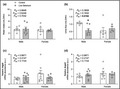

## 1 INTRODUCTION

A healthy diet during pregnancy is essential for maternal health and optimal fetal development, with maternal diets deficient in specific nutrients known to increase the risk of chronic disease in offspring as they age (Barker, 2007). Although many dietary elements are known to influence programed disease in offspring, the role of selenium remains poorly understood. Selenium is an essential trace element obtained from the diet that is required for the formation of selenocysteine, the 21^st^ proteinogenic amino acid (Hatfield et al., 2014). Selenocysteine is needed for the production of approximately 25 selenoproteins that are required for human health and are involved in processes such as antioxidant activity, mitochondrial function, and thyroid hormone metabolism (Papp et al., 2007). The key selenoproteins glutathione peroxidase (GPX) and thioredoxin reductase (TXNRD) are important antioxidant enzymes that protect tissues from oxidative stress. Given that 47% of healthy Australians do not meet the plasma selenium concentration required for optimal GPX activity (Lymbury et al., 2008), it is likely that many women would exhibit suboptimal levels of plasma selenium around the time of conception and during pregnancy. This is particularly concerning given the increased requirement for selenium throughout gestation. In Australia and New Zealand, the recommended daily intake of selenium is approximately 60 µg/day for nonpregnant women, with this increasing to 65 µg/day and 75 µg/day during pregnancy and lactation, respectively (Hofstee et al., 2018).

We have established a mouse model of maternal selenium deficiency which alters maternal thyroid hormone production and leads to fetal growth restriction (Hofstee et al., 2019). Offspring from this model develop impaired glucose homeostasis and thyroid hormone dysregulation by postnatal day 180 (PN180) (Hofstee, McKeating, et al., 2020). Interestingly, when we measured the mRNA expression of selenoproteins in fetal tissues, the kidney exhibited the greatest number of dysregulated selenoproteins including those involved in antioxidant function, mitochondrial biogenesis, and thyroid hormone metabolism (Hofstee et al., 2020a). This altered expression of antioxidant selenoproteins persisted in adulthood (Hofstee, Cuffe, et al., 2020). Given that the developing kidney is particularly vulnerable to the effects of adverse maternal diets (Dorey et al., 2014; Moritz et al., 2003), it is possible that suboptimal selenium during pregnancy may impede kidney development and lead to a functional impairment of this organ later in life. Although we have characterized the effects of maternal selenium deficiency on selenoprotein mRNA expression in the offspring kidney (Hofstee, Cuffe, et al., 2020), we have not explored how these changes affect seleno‐dependent antioxidant activity or redox status within this tissue. Although thyroid hormones are well known for their involvement in regulating metabolism, they also play a lesser known role in regulating kidney function (Mariani & Berns, 2012). Given that we have shown maternal selenium deficiency to alter plasma free thyroxine (T_4_) concentrations in adult offspring in a sex‐specific manner (increased in male offspring, decreased in female offspring) (Hofstee, McKeating, et al., 2020), it is possible that this altered T_4_ may impair offspring kidney parameters differently in males and females.

To our knowledge, the impact of maternal selenium deficiency on the offspring kidney has not been previously studied. Therefore, we aimed to investigate the influence of maternal selenium deficiency prior to and throughout pregnancy and lactation on kidney development and 24 hr urinary excretion in offspring. Given that programed disease often manifests in a sexually dimorphic manner, all analyses considered offspring sex as a factor.

## 2 METHODS

### 2.1 Mouse model of maternal selenium deficiency

This study utilized a mouse model of maternal selenium deficiency previously described (Hofstee et al., 2019). All animal work was completed with approval from Griffith University Animal Ethics Committee (MSC/01/16/AEC). The experimental design, animal housing, and animal husbandry were in accordance with the “Animals in Research: Reporting In Vivo Experiments” (ARRIVE) guidelines for DOHaD research (Dickinson et al., 2016). Briefly, female C57BL/6 mice were obtained from the Australian Resource Centre (ARC, Perth, Western Australia) and acclimatized for 1 week. Mice were randomly assigned and given *ad libitum* access to a control (>190 μg/kg) or low selenium (<50 μg/kg) diet for 4 weeks prior to mating, throughout gestation, and lactation. Mice were mated and monitored throughout pregnancy. Following birth, offspring were aged to adulthood (PN180). Mice were weaned at PN24. After weaning, mice were fed a selenium replete diet containing 230 μg Se/kg (Teklad Global 18% Protein Rodent Diet Irradiated, ENVIGO, WI, USA). To assess urinary excretion, mice were placed in individual metabolic cages for 24 hr at PN170 (*n* = 4–7 mice/group), with *ad libitum* access to food and water: 24 hr food and water intake were recorded, and urine was collected and stored at −30°C for subsequent urinalysis. One week later, mice were euthanized by cervical dislocation for plasma and kidney collection at PN180. Whole blood was collected via cardiac puncture into lithium heparin tubes (SARSTEDT, NRW, Germany), which were centrifuged at 2000 *g* for 5 min followed by collection of plasma. This plasma was used to measure T_4_ concentrations using a commercial ELISA (Invitrogen, CA, USA). This data have been previously published (Hofstee, McKeating, et al., 2020), but in this study is reanalyzed as a correlation with 24 hr urine flow. Kidneys were removed, decapsulated, snap frozen in liquid nitrogen, and stored at −80°C for subsequent molecular analysis. In all such molecular analyses, the whole kidney was crushed under liquid nitrogen to ensure all kidney cell types were represented in subsequent RNA, DNA, and protein extracts. A maximum of 1–2 males and 1–3 females were used from a single litter. This equated to the following minimum number of dams being used per group in each experiment: *n* = 5 control male, *n* = 5 low selenium female, *n* = 6 control female, and *n* = 3 low selenium female.

### 2.2 RNA extraction and quantitative PCR

RNA was extracted from 20 to 30 mg of crushed kidney tissue (*n* = 5‐8/group) using the RNeasy Mini Kit (Qiagen, VIC, Australia) according to the manufacturers protocol. RNA yield and purity were determined using a NanoDrop 2000/2000c Spectrophotometer (Thermo Fisher Scientific, MA, USA). RNA was reverse transcribed into cDNA using the iScript gDNA Clear cDNA Synthesis Kit (Bio‐Rad, CA, USA). Quantitative PCR was performed using the Quanti‐Nova SYBR Green PCR Kit (Qiagen, VIC, Australia) and predesigned KiCqStart SYBR Green primers (Sigma‐Aldrich, MO, USA) for superoxide dismutase 1 (*Sod1*), superoxide dismutase 2 (*Sod2*), aquaporin 2 (*Aqp2*), and arginine vasopressin receptor 2 (*Avpr2*) (Table 1). Briefly, 10 µL reactions were performed in duplicate and amplified using the QuantStudio 6 Real‐Time PCR system (Life Technologies, CA, USA). For each reaction, a melt curve was used to verify that only a single product had been amplified. Although a range of common housekeeping genes were trialled (*Gapdh*, *Actb*, *Rn18s*, *Sdha*), only *Gapdh* was found to be stably expressed across treatment groups and was selected for normalization. Expression was calculated by the 2^−ΔΔCt^ method compared to the expression of *Gapdh* and normalized to the mean of the male control group.

**TABLE 1:** qPCR primer list.

Role in kidney	Gene name	Gene acronym	Primer sequences
mRNA abundance			
Seleno‐independent antioxidants	Superoxide dismutase 1	*Sod1*	F′ CCAGTGCAGGACCTCATTTT R′ CACCTTTGCCCAAGTCATCT
	Superoxide dismutase 2	*Sod2*	F′ GGCCAAGGGAGATGTTACAA R′ GAACCTTGGACTCCCACA
Water balance	Aquaporin 2	*Aqp2*	F’ ACCTCCTTGGGATCTATTTC R’ GTAGTTGTAGAGGAGGGAAC
	Arginine vasopressin receptor 2	*Avpr2*	F’ CCAGGATAGGACCAATAGAC R’ TCCATGTGGAAAATGAACTG
Housekeeper	Glyceraldehyde 3‐phosphate dehydrogenase	*Gapdh*	F’ CCAGGAGCGAGACCCCACTAACA R’ TCGGCAGAAGGGGCGGAG
Mitochondrial content			
Mitochondrial DNA markers	Mitochondrial displacement loop^a^	*D‐loop*	F’ AATCTACCATCCTCCGTGAAACC R’ GCCCGGAGCGAGAAGAG
	NADH dehydrogenase 2	*Nd2*	F’ CACGATCAACTGAAGCAGCAA R’ ACGATGGCCAGGAGGATAATT
Nuclear DNA markers	Beta actin	*Actb*	F’ AGCCATGTACGTAGCCATCCA R’ TCTCCGGAGCCATCACAATG
	Glyceraldehyde 3‐phosphate dehydrogenase	*Gapdh*	F’ AAGGTCATCCCAGAGCTGAA R’ CTGCTTCACCACCTTCTTGA

^a^ Non‐coding region of mitochondrial DNA.

### 2.3 Mitochondrial content

DNA was extracted from 20 to 30 mg of crushed kidney tissue (*n* = 6‐8/group) using the DNeasy Blood and Tissue Kit (Qiagen, VIC, Australia). qPCR was used to amplify DNA using primer sets for two mitochondrial‐encoded DNA markers (*NDA2‐NADH dehydrogenase 2* and *D*‐*loop*) and two nuclear‐encoded DNA markers (*Actb* and *Gapdh*). Geometric means for the mitochondrial and nuclear makers were calculated. Relative mitochondrial content was calculated by the 2^−ΔΔCt^ method (ratio of mitochondrial to nuclear DNA) and normalized to the mean of the male control group.

### 2.4 Protein extraction and estimation

Protein was extracted from 20 to 30 mg of crushed kidney tissue (*n* = 5‐8/group) in 300 µL RIPA buffer using an IKA T 10 basic ULTRA‐TURRAX hand‐held homogenizer (IKA, NRW, Germany). Protein yield was estimated using an RC DC protein assay (Bio‐Rad, CA, USA). Protein samples were diluted 1:10 and added to a 96 well plate with pre‐prepared protein standards ranging from 0 µg/µL to 2 µg/µL. Unknown protein concentrations were calculated from the standard curve and corrected for the 1:10 dilution. All protein samples were normalized to a final concentration of 3 µg/µL.

### 2.5 Glutathione peroxidase and thioredoxin reductase activity

Total glutathione peroxidase (GPX) and thioredoxin reductase (TXNRD) activities were measured in *n* = 5–8 samples per group using commercially available kits according to the manufacturer's protocols (Caymen Chemical, MI, USA). For each assay, 1 µg/µL of protein diluted in water was used, with absorbance measured at 340 nm for GPX activity and 405 nm for TXNRD activity. Activity levels were calculated after correcting for background, with all samples run in triplicate in a single assay. The intraplate coefficient of variation was 0.83% for GPX activity and 6.26% for TXNRD activity.

### 2.6 Hydrogen peroxide assay

Fresh standards ranging from 10 μM to 0.02 μM hydrogen peroxide (H_2_O_2_) were prepared in molecular grade water. A reaction mix containing Amplex Ultra Red (Invitrogen, CA, USA), superoxide dismutase (SOD), and horseradish peroxidase (HRP) was prepared and added to samples and standards loaded into a 96‐well plate. Following a 30 min incubation, fluorescence was measured (excited at 545 nm, detected at 587 nm) and H_2_O_2_ concentrations calculated from the standard curve. In each group, *n = *5–8 samples were assayed in duplicate in a single assay. The intraplate coefficient of variation was 2.88%.

### 2.7 Advanced glycation end product (AGE) assay

Protein glycation was assessed using the Glycated Protein Detection Kit (Abcam, Cambridge, UK). *n = *5–6 protein samples per group were labeled with fluorescein–boronic acid according to the manufacturer's protocol. Labeled proteins were denatured and 9 μg of total protein loaded onto 12% polyacrylamide gels for separation by SDS‐PAGE. Male and female samples were run on separate gels and imaged under blue light using a Chemi‐Doc MP Imaging System (Bio‐Rad, CA, USA). Proteins were then transferred to immunoblot low fluorescence polyvinylidene difluoride membranes (Bio‐Rad, CA, USA) at 90 V for 1 hr, incubated with Revert 700 Total Protein Stain (Li‐Cor Biosciences, NE, USA) according to the recommended protocol, and imaged at 700 nm to detect total protein. Densitometry values were calculated from the entire lane using Image Studio Lite (v5.2) software (Li‐Cor Biosciences, NE, USA). Relative AGE concentrations were calculated by normalizing the densitometry values obtained from the AGE assay to the values of the total protein loading control for each sample.

### 2.8 Western blotting

Western blotting was performed by loading 11.25 µg of protein into 15‐lane 12% resolving gels. All male samples were loaded onto one gel and all female samples loaded onto a separate gel and subjected to SDS–PAGE. To identify protein size, 2 µL of Precision Plus Protein Dual Colour Standards (Bio‐Rad, CA, USA) were loaded. Gels were run at 90 V for 2.5 hr. Proteins were transferred to immunoblot low fluorescence polyvinylidene difluoride membranes (Bio‐Rad, CA, USA) at 90 V for 1 hr. Membranes were blocked in Odyssey Blocking Buffer (PBS) (Li‐Cor Biosciences, NE, USA) for 1–2 hr at room temperature. Membranes were incubated in Total OXPHOS Rodent WB Antibody Cocktail (Abcam; ab110413; 1:1000) followed by IRDye 800CW donkey anti‐mouse IgG (Li‐Cor; 1:15,000) both for 1 hr. In between all incubation steps, membranes were washed thoroughly in PBS with 10% Tween on an Orbital Shaker (Alkali Scientific, FL, USA). Membranes were scanned using an Odyssey CLx fluorescent scanner (Li‐Cor Biosciences, NE, USA) and quantified in the Image Studio Lite (v5.2) software provided by the manufacturer. For each sample, the densitometry values were obtained for a subunit of complex II, III, IV, and V. These bands were normalized to the value for the total protein loading control (as per AGE assay). Blots were repeated, with the average value for each sample used for analysis. Complex I could not be reliably detected and was therefore not quantified.

### 2.9 Urinalysis

Total urine volume collected in the metabolic cage was measured to determine 24 hr urine flow. Urinary sodium, potassium, chloride, glucose, urea, albumin, creatinine and total protein were measured using an Integra 400 plus automated chemistry analyzer (Roche Diagnostics, NSW, Australia). Urine sample volume used was 200 μL per sample from *n* = 4–7 mice per group. All assays were performed using assay kits from the manufacturer and were calibrated using Calibrator for Automated Systems reagent. Quality control was performed using PreciControl ClinChem Multi 1 and 2 (Roche Diagnostics, NSW, Australia) prior to sample analysis. All samples were analyzed within a single run.

### 2.10 Statistical analysis

Statistical analyses were performed in GraphPad Prism 9.0 (GraphPad Software, CA, USA). Data are represented as mean ± SEM. All data were assessed for normality using the Shapiro–Wilk normality test prior to being analyzed by two‐way analysis of variance (ANOVA) with the main effects of maternal selenium deficiency (*P*_trt_) and sex (*P*_sex_), and any interaction between these factors (*P*_int_), examined. If a significant main effect was detected (*P* < 0.05), this was followed by Sidak's *post hoc* multiple comparisons test to compare the low selenium group to the control group within each sex. When normality was not met, a Box‐Cox transformation was first applied prior to statistical analysis. For AGE quantification and Western blotting, data were analyzed separately by sex using unpaired *t* test (with Welch correction if required). If non‐normal, data were instead analyzed by Wilcoxon rank‐sum test. Correlations between urine flow and plasma T_4_ were assessed by Pearson correlation if normal or Spearman correlation if non‐normal. For all analyses, statistical significance was set at *P* < 0.05. When multiple mice of the same sex were used from a single litter, data were not averaged and were treated independently to maintain adequate statistical power.

## 3 RESULTS

### 3.1 Allometry

At PN180, maternal selenium deficiency had no impact on offspring body weight, kidney weight, or kidney weight to body weight ratio (Table 2). Female offspring had a lighter body weight (*P_sex_* < 0.0001) and kidney weight (*P_sex_* < 0.0001) and a higher kidney‐to‐body weight ratio (*P_sex_ *= 0.0417) compared with male offspring.

**TABLE 2:** The effects of maternal selenium deficiency on offspring allometry.

Parameter	Male		Female		Two‐way ANOVA		
	Control	Low Selenium	Control	Low Selenium	*P*_trt_	*P*_sex_	*P*_int_
*n*	7	8	8	6			
BW (g)	28.18 (1.00)	28.34 (1.22)	21.68 (0.40)	20.91 (0.39)	0.7351	<**0.0001**	0.6018
KW (mg)	181.2 (6.6)	172.8 (7.9)	126.0 (4.30)	122.3 (1.24)	0.3200	<**0.0001**	0.6955
KW/BW (mg/g)	0.156 (0.003)	0.166 (0.008)	0.173 (0.004)	0.171 (0.003)	0.4575	**0.0417**	0.2866

Notes

Abbreviations: BW, body weight. KW, kidney weight. KW/BW, kidney‐to‐body weight ratio.

Data are mean (SEM). Kidney weight calculated as the average weight of the left and right kidney. Data analyzed by two‐way ANOVA with Sidak's *post hoc*. Significant main effects are indicated in bold. No differences were found between control and low selenium groups. Significance determined at *P* < 0.05.

### 3.2 Antioxidant activity and gene expression

**FIGURE 1:**
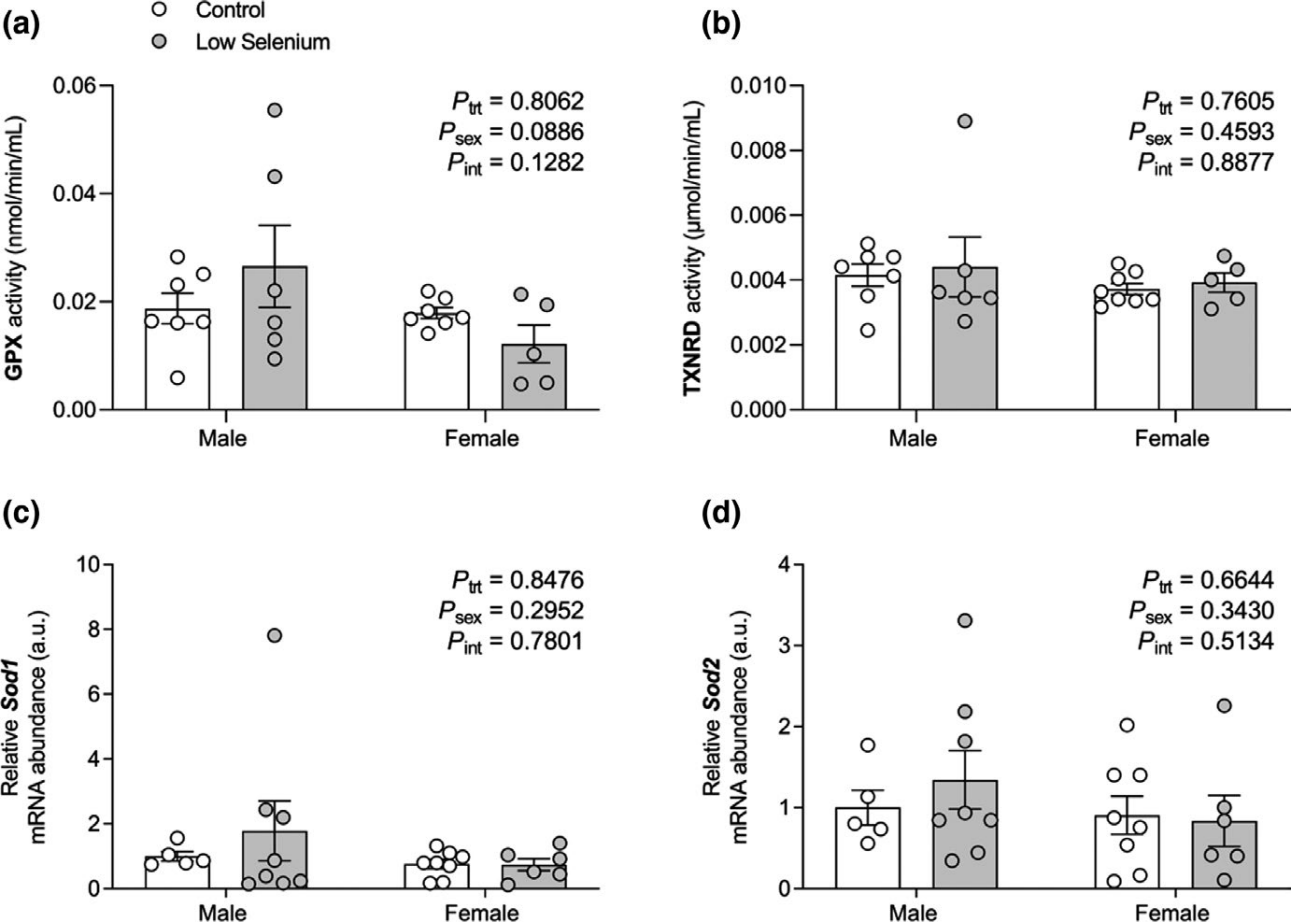
The effects of maternal selenium deficiency on seleno‐dependent antioxidant activity and non‐seleno‐dependent gene expression in the offspring kidney. (a) GPX protein activity (*n* = 7 control male, *n* = 6 low selenium male, *n* = 7 control female, *n* = 5 low selenium female), (b) TXNRD protein activity (*n* = 7 control male, *n* = 6 low selenium male, *n* = 8 control female, *n* = 5 low selenium female), (c) *Sod1* mRNA abundance (*n* = 5 control male, *n* = 8 low selenium male, *n* = 8 control female, *n* = 6 low selenium female) and (d) *Sod2* mRNA abundance (*n* = 5 control male, *n* = 8 low selenium male, *n* = 8 control female, *n* = 6 low selenium female) in the kidneys of male and female offspring mice (PN180) exposed to a control (open circle) or low selenium (closed circle) maternal diet. Data are mean ±SEM. Data analyzed by two‐way ANOVA with Sidak's *post hoc*. Significance determined at *P* < 0.05. For TXNRD activity and *Sod1* mRNA, data were Box‐Cox transformed prior to statistical analyses but non‐transformed data is presented.

As *Txnrd* mRNA levels were increased within the kidney at PN180 following maternal selenium deficiency in our recent publication (Hofstee, Cuffe, et al., 2020), it was important to explore GPX and TXNRD activity in the current study. However, maternal selenium deficiency did not alter GPX or TXNRD activity (Figure 1a and 1b). As these seleno‐dependent antioxidants adapted to maternal selenium deficiency, we examined if this altered the non‐seleno‐dependent antioxidants superoxide dismutases 1 and 2 (*Sod1*/*2)*. *Sod1* and *Sod2* mRNA were unaffected by maternal selenium deficiency (Figure 1c and 1d).

### 3.3 Markers of oxidative stress

**FIGURE 2:**
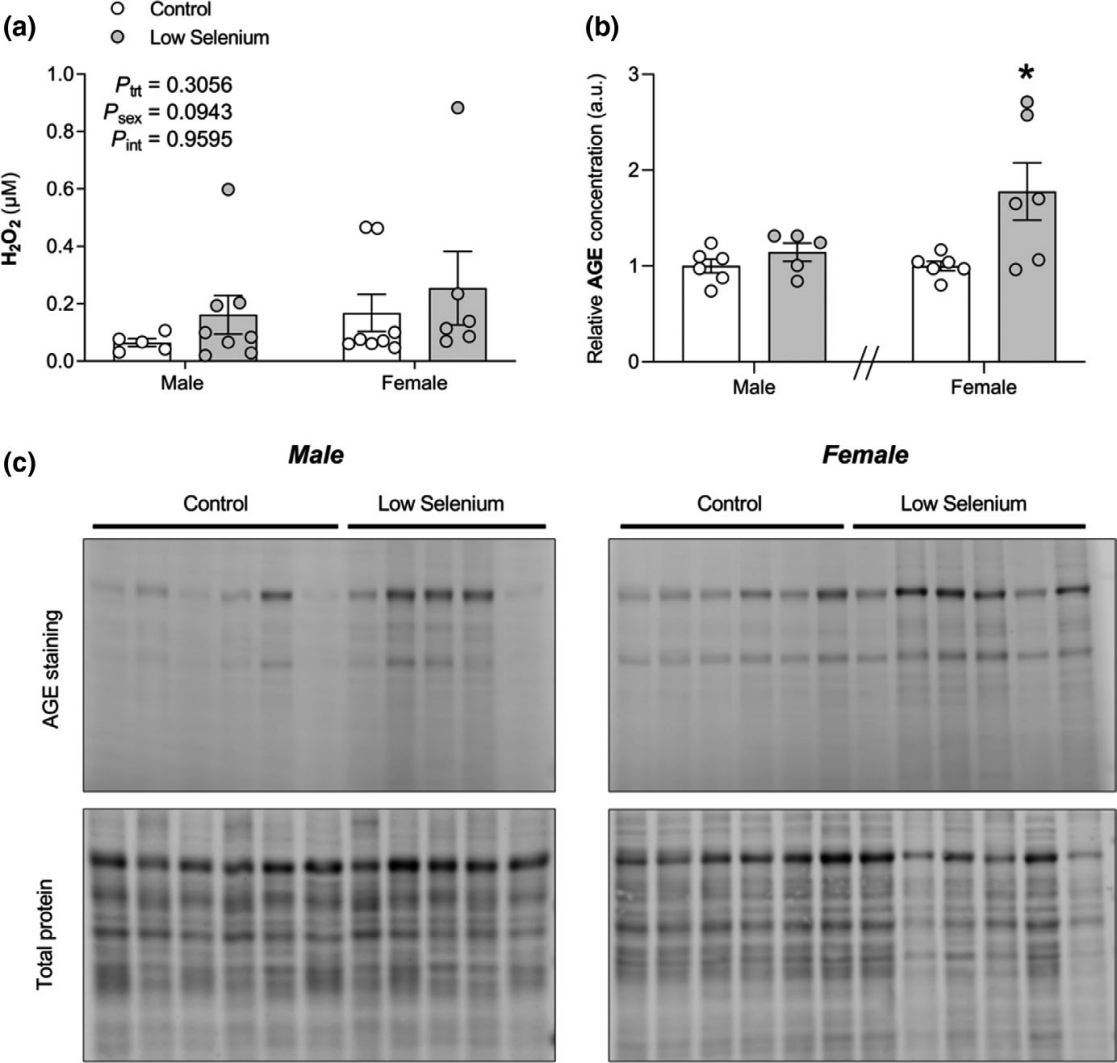
The effects of maternal selenium deficiency on markers of oxidative stress in the offspring kidney. (a) Hydrogen peroxide (H_2_O_2_) concentrations (*n* = 5 control male, *n* = 8 low selenium male, *n* = 8 control female, *n* = 6 low selenium female) and (b) relative advanced glycation end product (AGE) concentrations (*n* = 6 control male, *n* = 5 low selenium male, *n* = 6 control female, *n* = 6 low selenium female) in the kidneys of male and female offspring mice (PN180) exposed to a control (open circle) or low selenium (closed circle) maternal diet. AGE gels with total protein loading controls quantified in (b) are shown in (c). Data are mean ±SEM. H_2_O_2_ data analyzed by two‐way ANOVA with Sidak's *post hoc*. These data were Box‐Cox transformed prior to statistical analysis but non‐transformed data is presented. AGE data were split by sex and analyzed by unpaired *t*‐test with Welch correction. *P < 0.05. Significance determined at *P* < 0.05.

To verify that the function of these seleno‐dependent antioxidant systems had normalized by PN180, we measured markers of oxidative stress in the kidney. Maternal selenium deficiency did not alter concentrations of hydrogen peroxide (H_2_O_2_) (Figure 2a). Although AGE concentrations were not different between low selenium males and control males (*P* = 0.2500, Figure 2b), they were 78% higher in low selenium females compared with control females (*P* = 0.0485, Figure 2b).

### 3.4 Markers of mitochondrial function

**FIGURE 3:**
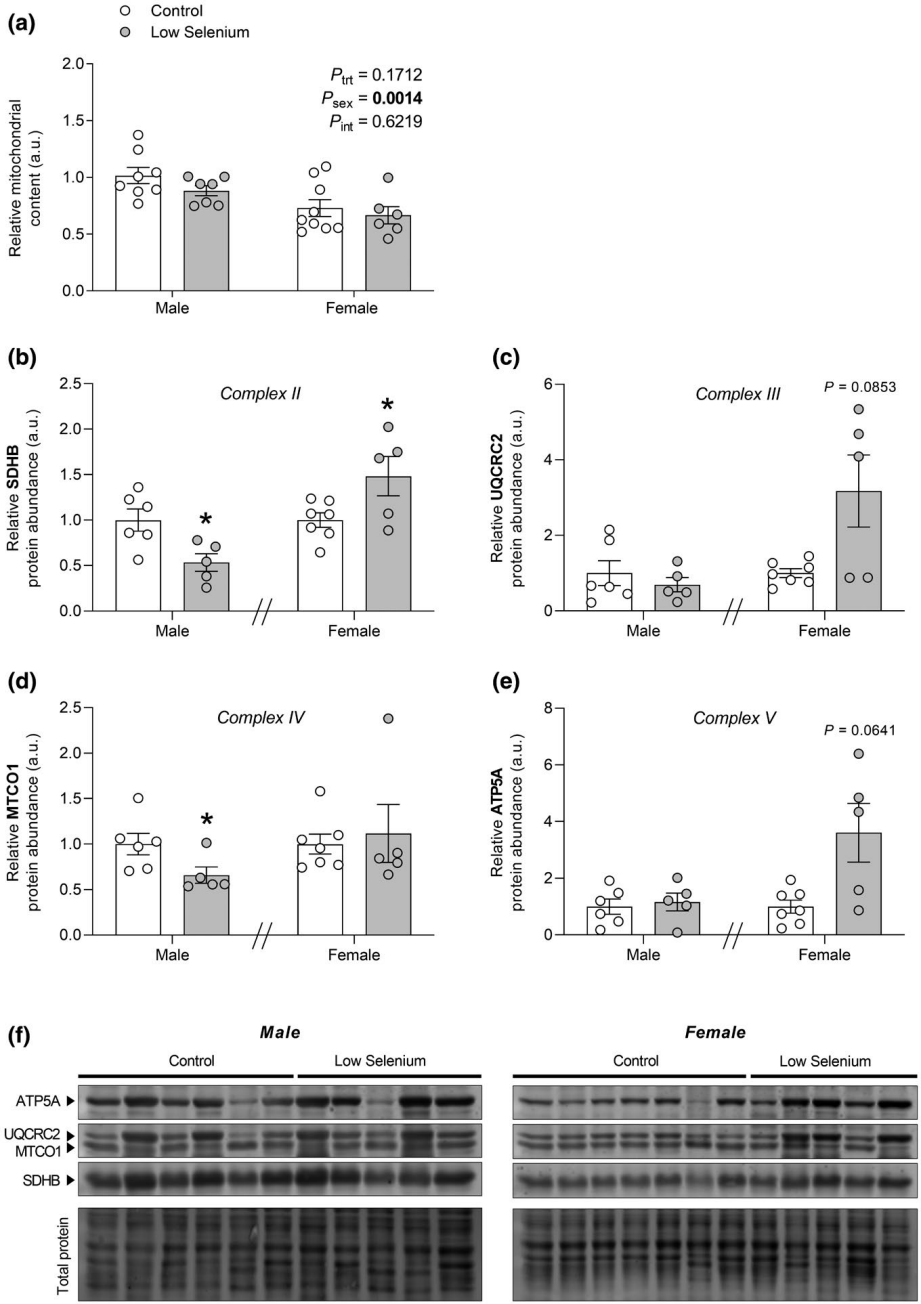
The effects of maternal selenium deficiency on markers of mitochondrial function in the offspring kidney. (a) Mitochondrial content (*n* = 8 control male, *n* = 7 low selenium male, *n* = 8 control female, *n* = 6 low selenium female), (b) SDHB protein abundance (*n* = 6 control male, *n* = 5 low selenium male, *n* = 7 control female, *n* = 5 low selenium female), (c) UQCRC2 protein abundance (*n* = 6 control male, *n* = 5 low selenium male, *n* = 7 control female, *n* = 5 low selenium female), (d) MTCO1 protein abundance (*n* = 6 control male, *n* = 5 low selenium male, *n* = 7 control female, *n* = 5 low selenium female) and (e) ATP5A protein abundance (*n* = 6 control male, *n* = 5 low selenium male, *n* = 7 control female, *n* = 5 low selenium female) in the kidneys of male and female offspring (PN180) exposed to a control (open circle) or low selenium (closed circle) maternal diet. Data are mean ±SEM. Mitochondrial content analyzed by two‐way ANOVA with Sidak's *post hoc*. Male samples and female samples were run on separate gels for Western blotting and therefore analyzed independently. Western blots were analyzed by unpaired *t*‐test with Welch correction if required except for male MTCO1 data, which was non‐normally distributed and was analyzed by Wilcoxon rank‐sum test. *P < 0.05. Significance determined at *P* < 0.05. In (b‐e), data averaged from two replicates with a single blot shown for each sex in (f).

Given that AGE accumulation has been linked to mitochondrial dysfunction, we examined markers of mitochondrial function in the kidney. Selenium deficiency did not modify mitochondrial content (Figure 3a), although there was an effect of sex, with females exhibiting lower mitochondrial content compared with males (*P*_sex_ = 0.0014, Figure 3a). Complex II (succinate dehydrogenase complex iron sulfur subunit B, SDHB) protein abundance was altered by selenium deficiency in a sexually dimorphic manner. Low selenium males exhibited a 47% decrease compared with control males (*P* = 0.0171, Figure 3b), whereas low selenium females exhibited a 48% increase compared with control females (*P* = 0.0381, Figure 3b). Complex IV (mitochondrially encoded cytochrome C oxidase I, MTCO1) protein abundance was reduced by 34% in low selenium males compared with control males (*P* = 0.0303, Figure 3d), but was unchanged in females (*P* = 0.7551, Figure 3d). Complex III (ubiquinol‐cytochrome C reductase core protein 2, UQCRC2, Figure 3c) and complex V (ATP synthase F1 subunit alpha, ATP5A, Figure 3e) protein abundance were not affected by maternal selenium deficiency in either sex.

### 3.5 Metabolic parameters and expression of genes regulating water reabsorption

**FIGURE 4:**
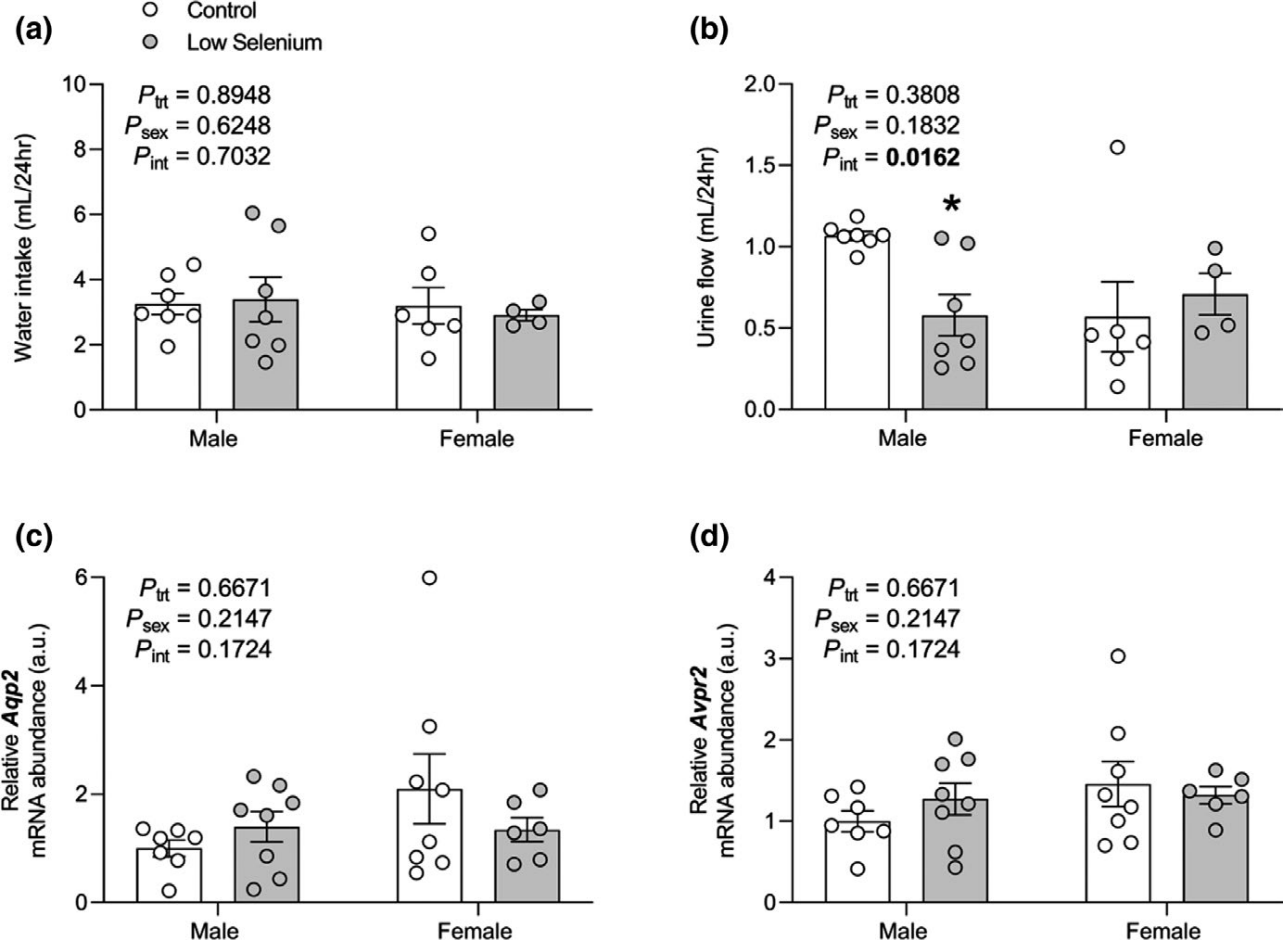
The effects of maternal selenium deficiency on 24 hr water intake and urine flow and associated gene expression in the offspring kidney. (a) 24 hr water intake (*n* = 7 control male, *n* = 7 low selenium male, *n* = 7 control female, *n* = 4 low selenium female), (b) 24hr urine flow (*n* = 7 control male, *n* = 7 low selenium male, *n* = 6 control female, *n* = 4 low selenium female), (c) *Aqp2* mRNA abundance (*n* = 7 control male, *n* = 8 low selenium male, *n* = 8 control female, *n* = 6 low selenium female) and (d) *Avpr2* mRNA abundance (*n* = 7 control male, *n* = 8 low selenium male, *n* = 8 control female, *n* = 6 low selenium female) in male and female offspring mice (PN180) exposed to a control (open circle) or low selenium (closed circle) maternal diet. Data are mean ±SEM. Data analyzed by two‐way ANOVA with Sidak's *post hoc*. *P < 0.05 between control and low selenium groups of the same sex according to *post hoc* analysis. Significance determined at *P* < 0.05. For 24 hr urine flow, data were Box‐Cox transformed prior to statistical analysis but non‐transformed data is presented.

Maternal selenium deficiency had no effect on 24 hr food intake (Figure S1) or 24 hr water intake (Figure 4a). However, we found a significant interaction between treatment and sex for 24 hr urine flow (*P_int_* = 0.0162), with *post hoc* analysis revealing that this was driven by a 53% reduction in urine flow in low selenium males compared with control males (*P* = 0.0239, Figure 4b). There was no difference in urine flow in low selenium females compared with control females (*P* = 0.2746, Figure 4b). To investigate a possible mechanism through which maternal selenium deficiency may be reducing 24 hr urine flow in male offspring, we quantified the mRNA abundance of aquaporin 2 (*Aqp2*) and arginine vasopressin receptor 2 (*Avpr2*) due to their important role in regulating water reabsorption within the collecting duct. However, neither *Aqp2* (Figure 4c) nor *Avpr2* (Figure 4d) mRNA were affected by maternal selenium deficiency.

### 3.6 Urinary electrolytes and proteins

Maternal selenium deficiency did not impact the urinary concentrations of sodium, potassium, chloride, glucose, urea, albumin, creatinine, or total protein (Table 3). Urinary glucose concentrations were higher in female offspring compared with male offspring (*P_sex_* = 0.0298), whereas urinary total protein concentrations were lower in male offspring compared with female offspring (*P_sex_* = 0.0001).

**TABLE 3:** The effects of maternal selenium deficiency on urinary electrolyte, glucose and protein concentrations in offspring.

Parameter	Male		Female		Two‐way ANOVA		
	Control	Low Selenium	Control	Low Selenium	*P*_trt_	*P*_sex_	*P*_int_
*n*	7	7	6	4			
Sodium (mmol/L)	105.4 (4.5)^b^	139.7 (22.8)	141.6 (16.1)	129.5 (13.1)	0.5339	0.4687	0.2029
Potassium (mmol/L)^a^	189.2 (16.9)^b^	232.5 (38.0)	261.8 (20.2)	235.3 (10.3)	0.9868	0.0897	0.6292
Chloride (mmol/L)	133.4 (11.4)^b^	166.8 (26.3)	190.6 (12.2)	183.4 (16.1)	0.5138	0.0756	0.3144
Glucose (mmol/L)^a^	2.20 (0.15)	4.78 (1.65)	4.10 (0.38)	3.52 (0.29)	0.9062	**0.0298**	0.6912
Urea (mmol/L)^a^	1301 (120)	1696 (350)	1993 (169)	1737 (112)	0.8658	0.0949	0.7317
Albumin (mg/L)	18.21 (1.70)	15.11 (0.67)	15.18 (0.79)	16.87 (0.21)	0.5573	0.5945	0.0564
Creatinine (mmol/L)	3.74 (0.19)	4.83 (0.79)	5.36 (0.71)	4.46 (0.53)	0.8837	0.3364	0.1298
Total protein (mg/L)	5757 (410)	6453 (1210)	2395 (642)	1824 (404)	0.9423	**0.0001**	0.4665

Data are mean (SEM). Data analyzed by two‐way ANOVA with Sidak's *post hoc*. Significant main effects are in bold. No differences were found between control and low selenium groups for either sex for any parameter examined by *post hoc* analysis (all *P* > 0.05). Significance determined at *P* < 0.05.

^a^ Data were Box‐Cox transformed prior to statistical analyses but non‐transformed data is presented.

^b^ *n* = 6 due to technical error.

### 3.7 Thyroid hormone regulation of renal function

**FIGURE 5:**
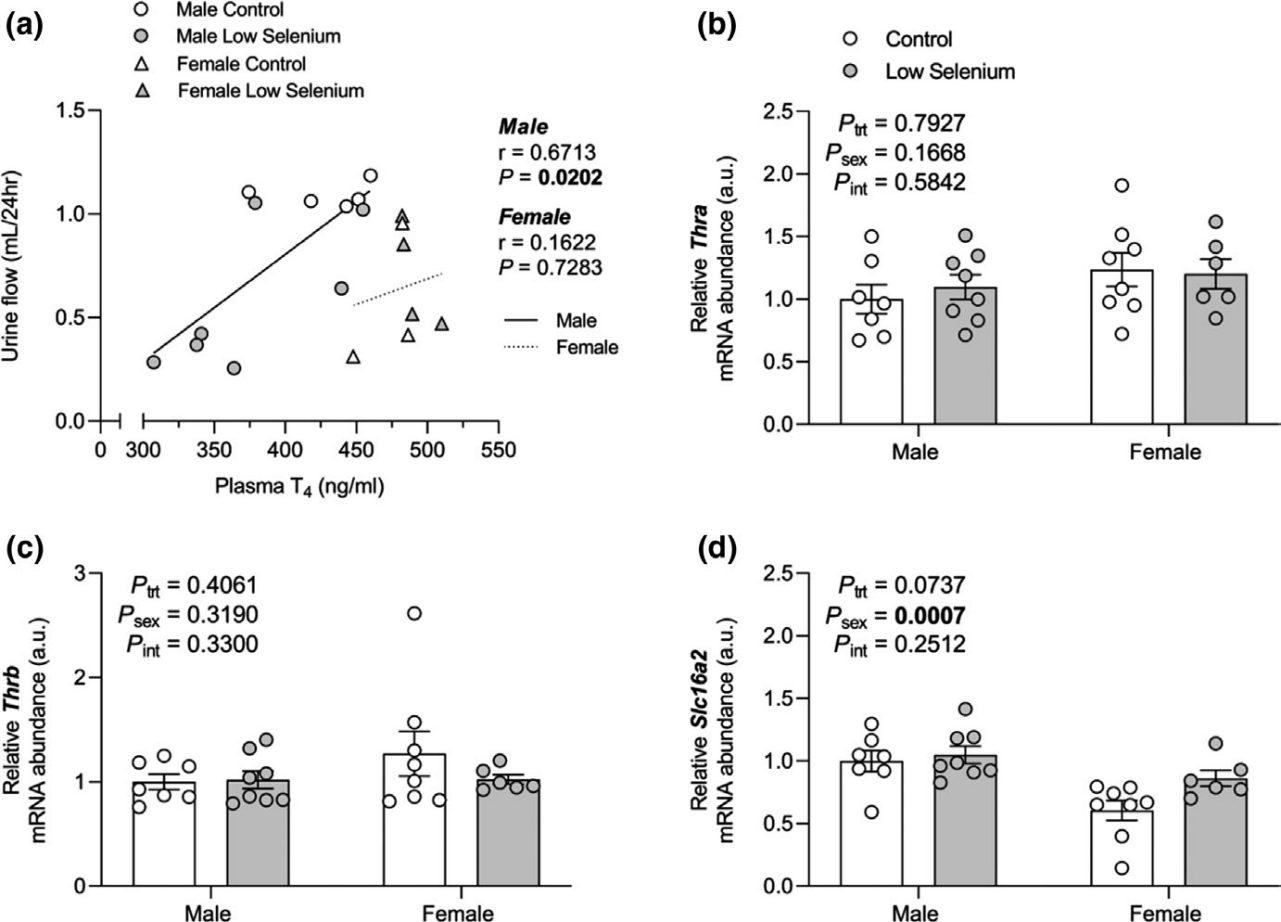
The effects of maternal selenium deficiency on thyroid hormone signalling in the offspring kidney. (a) Correlations between 24hr urine flow and plasma T_4_ in male (circle, *n* = 5 control, *n* = 7 low selenium) and female (triangle, *n* = 3 control, *n* = 4 low selenium) control (open symbol) and low selenium (closed symbol) offspring. (b) *Thra* mRNA abundance (*n* = 7 control male, *n* = 8 low selenium male, *n = *8 control female, *n* = 6 low selenium female), (c) *Thrb* mRNA abundance (*n* = 7 control male, *n* = 8 low selenium male, *n = *8 control female, *n* = 6 low selenium female) and (d) *Slc16a2* mRNA abundance (*n* = 7 control male, *n* = 8 low selenium male, *n = *8 control female, *n* = 6 low selenium female) in male and female offspring mice (PN180) exposed to a control (open circle) or low selenium (closed circle) maternal diet. Data are mean ±SEM. For correlations, female data were analyzed by Pearson correlation and male data by Spearman correlation. All other data were analyzed by two‐way ANOVA with Sidak's *post hoc*. No differences were found between control and low selenium groups for either sex for any parameter examined by *post hoc* analysis (all *P* > 0.05). Significance determined at *P* < 0.05. For *Slc16a2* mRNA, data were Box‐Cox transformed prior to statistical analysis but non‐transformed data is presented.

Many studies have demonstrated relationships between thyroid hormone status and markers of urinary excretion. Plasma T_4_ positively correlated with 24 hr urine flow in male offspring (r = 0.6713, *P* = 0.0202, Figure 5a), but not in female offspring (r = 0.1622, *P* = 0.7283, Figure 5a). The mRNA abundance of thyroid receptor alpha (*Thra*), thyroid receptor beta (*Thrb*), and monocarboxylate transporter 8 (*Slc16a2*) was not altered by maternal selenium deficiency (Figure 5b–5d).

## 4 DISCUSSION

The effects of maternal selenium deficiency on offspring kidney parameters have not previously been explored. We have previously demonstrated that women with low selenium have altered thyroid function and an increased risk of developing a range of poor pregnancy outcomes (Hofstee, James‐McAlpine, et al., 2020). Given that adverse pregnancy outcomes can predispose offspring to disease, we established a mouse model of selenium deficiency in pregnancy. We have recently demonstrated in this model that maternal selenium deficiency induces fetal growth restriction (Hofstee et al., 2019), alters selenoprotein expression (Hofstee, Cuffe, et al., 2020), and perturbs an array of metabolic parameters in the mother and fetus (Hofstee et al., 2019) and in 6‐month offspring (Hofstee, McKeating, et al., 2020). Given that the kidney has been shown to be vulnerable to the detrimental effects of an adverse prenatal environment, we hypothesized that these alterations to fetal growth and metabolism caused by maternal selenium deficiency would lead to impairment of the kidney in later life. Indeed, we observed that a maternal diet low in selenium resulted in sex‐specific alterations to kidney parameters related to tissue glycation, mitochondrial protein expression, and urine flow. Importantly, this study suggests that these changes in kidney glycation, mitochondrial protein expression, and urine flow are not linked to deficits in renal antioxidant activity. Therefore, the changes to kidney parameters we report on in the present study are likely linked to the previously reported thyroid dysfunction and hyperglycemia that is characteristic of this model (Hofstee et al., 2019; Hofstee, McKeating, et al., 2020). This study highlights that selenium deficiency during pregnancy can have long‐term consequences for offspring kidney health in adult life.

As elevated oxidative stress is known to perturb renal function, we investigated antioxidant activity and markers of oxidative stress in the kidneys from offspring exposed to maternal selenium deficiency. A key finding from one of our recent publications is that selenium deficiency increased the mRNA expression of 14 selenoproteins in the fetal kidney (Hofstee, Cuffe, et al., 2020). Although the mRNA expression of most of these selenoproteins returned to levels similar to controls by PN180, the expression of two key selenium‐dependant antioxidants, *Txnrd1* and *Txnrd2*, remained elevated. In the current study, we demonstrate that this long‐term increase in *Txnrd1* and *Txnrd2* mRNA does not result in increased enzymatic activity of TXNRD, or the other major selenium‐dependant antioxidant, GPX. These findings could suggest that the kidney is increasing *Txnrd* mRNA to maintain enzymatic activity in the face of maternal selenium deficiency to combat long‐term deficits in adult life. Next, we examined if non‐seleno‐dependent antioxidants were dysregulated in the kidneys of offspring exposed to maternal selenium deficiency and demonstrated no changes in *Sod1* or *Sod2* mRNA. This aligns with our finding that maternal selenium deficiency does not alter H_2_O_2_ concentrations, a marker of oxidative stress, in the offspring kidney.

Although H_2_O_2_ concentrations were not affected by maternal selenium deficiency, we also characterized the relative concentrations of AGEs in the kidney, which are formed as a consequence of concurrent oxidation and non‐enzymatic glycation of proteins and lipids (Bohlender et al., 2005). AGE concentrations were increased in the kidneys of female but not male offspring of low selenium dams. Considering that markers of antioxidant function and oxidative stress were normal in low selenium females, we attribute this increase in AGEs to the poor long‐term glycemic control exhibited by these mice. It is well‐established that the degree of kidney glycation is dependent on the severity and duration of hyperglycemia (Liu et al., 2018). Although it is unclear the age at which female low selenium mice developed hyperglycemia in this cohort, at PN170, the female low selenium mice reported on in this study had significantly poorer glucose homeostasis than female controls based on glucose tolerance testing performed previously (Hofstee, McKeating, et al., 2020). Given that glucose intolerance was not apparent in male low selenium mice, this likely explains why AGE levels were not increased in this group in this study. AGEs are ligands for the receptor for AGEs (RAGE), which is expressed by podocyte and endothelial cells of the kidney (Busch et al., 2010). Activation of RAGE by AGEs can trigger apoptosis and promote proinflammatory cytokine production through upregulation of transcription factor nuclear factor‐kappaB, or NF‐κB (Ott et al., 2014). Given this, it is important that future studies interrogate if maternal selenium deficiency leads to heightened activation of the AGE/RAGE axis in the kidneys of female offspring.

Previous studies have demonstrated that various prenatal insults can dysregulate mitochondrial DNA content in fetal and offspring tissue. Maternal iron deficiency has been demonstrated to reduce mitochondrial content in the kidneys of male but not female fetuses (Woodman et al., 2018). In the current study, maternal selenium deficiency did not impact mitochondrial content in the offspring kidney; however, the abundance of several proteins involved in the electron transport chain were altered in a sex‐specific manner. SDHB (a component of Complex II) and MTCO1 (a component of Complex IV) were reduced in low selenium males, whereas SDHB was increased in low selenium females. Given that these changes were not accompanied by alterations to mitochondrial DNA content (a crude indicator of mitochondrial number), this suggests that changes in the abundance of proteins involved in the electron transport chain are not driven simply by alterations to the total number of mitochondria within the kidney. It is possible that such changes may therefore indicate changes in mitochondrial function, such as an altered capacity to undergo oxidative phosphorylation. However, given that our study did not assess functional parameters such as citrate synthase activity or mitochondrial respiration, it is unclear if such changes are linked to altered mitochondrial function. Despite this, it is likely that such changes are driven by sex‐specific alterations in thyroid hormone metabolism.

Recently, we have shown that maternal selenium deficiency increases T_4_ concentrations in pooled fetal plasma (Hofstee et al., 2019). We also found in the same cohort of offspring analyzed in the current study that T_4_ concentrations were lower in male offspring of low selenium dams and higher in female offspring of low selenium dams compared with control offspring of the same sex (Hofstee, McKeating, et al., 2020). Thyroid hormone is known to regulate many aspects of metabolism, including the expression and activity of mitochondrial complexes (Louzada & Carvalho, 2018). In particular, T_3_ administration has been shown to increase the expression of all of the mitochondrial complexes and the activity of Complex I and Complex II (Silvestri et al., 2018). As such, the sex‐specific differences in Complex II and Complex IV are likely driven by the different thyroid hormone responses in male and female offspring. Therefore, maternal selenium deficiency may alter mitochondrial parameters within the offspring kidney in a T_4_‐dependent manner. Similarly, thyroid hormone is known to be important for kidney development (Forhead & Fowden, 2014), and so the selenium deficiency induced increase in fetal thyroid hormone previously published in this model (Hofstee et al., 2019) may have contributed to the renal deficits seen in the current study. Given the abundant literature demonstrating that glucocorticoids also regulate kidney development (Forhead et al., 2015; Moritz et al., 2011; O'Sullivan et al., 2015), we also assessed maternal corticosterone in our previous cohort (Hofstee et al., 2019). Importantly, maternal and offspring corticosterone levels were unaffected by maternal selenium deficiency, suggesting the long‐term renal deficits in the current study are more strongly linked to alterations in thyroid hormone than corticosterone. To ascertain if maternal selenium deficiency has programed offspring disease by altering maternal thyroid function, future animal studies are required. Such studies may involve investigating the direct effects of maternal hyperthyroidism on offspring kidney function or determining if restoration of maternal thyroid status prevents long‐term disease.

AGE accumulation within the kidney is associated with increased cross‐linking of extracellular matrix proteins and basement membrane collagen deposition (Cohen et al., 1995; Vlassara et al., 1994). Forbes *et al*. (2003) have shown the accumulation of AGEs in the kidneys of STZ‐induced diabetic rats to be associated with structural impairment and an increased abundance of transforming growth factor beta 1 and collagen IV within this organ. With this considered, we expected increased AGEs within the kidneys of low selenium females to manifest in impaired urinary excretion; however, urinary excretion in this group was found to be normal. Contrary to what was anticipated, aberrant urinary excretion was identified in male offspring, not female offspring. More specifically, this group displayed a marked reduction in 24 hr urine flow compared with controls of the same sex, despite no difference in water intake during this period. This reduced urine flow in males was not linked with changes in urinary electrolyte, protein, or glucose concentrations. However, given that urine flow was reduced to approximately half that of control levels in low selenium male offspring, the total excretion of such analytes over a 24 hr period would be reduced, likely resulting in their accumulation within plasma which could be potentially harmful. However, due to low sample availability, we were unable to measure these analytes within plasma and thus this cannot be confirmed in this study. Modified *Aqp2* and *Avpr2* expression are commonly reported alongside alterations to urine flow (Dorey et al., 2019; Nejsum et al., 2001), but in the kidneys of low selenium male offspring, these mRNA were unchanged. Future studies need to examine AQP2 protein abundance and sub‐cellular localization to determine if the trafficking of this water channel has been affected. As AQP2 activity is highly dependent upon its phosphorylation status, phosphorylated AQP2 protein abundance should also be measured (Gooch et al., 2006) along with plasma vasopressin, an activator of AQP2.

An interesting finding from the current study is that urine flow correlated with plasma T_4_ concentrations, but only in male offspring. It is widely accepted that thyroid hormones play a major role in the regulation of water excretion as made evident by hypothyroid patients having a reduced ability to excrete a water load (reviewed by McDonald et al. (1976)). This is thought to be due to dysfunction at multiple levels, including reduced cardiac output, reduced renal blood flow, and therefore lower glomerular filtration rate (Rhee, 2016). Studies have demonstrated that thyroid hormones can influence sodium transport in the proximal tubule of the kidney. Thyroid hormones mediate this effect through increasing Na^+^/K^+^‐ATPase transcription (McDonough et al., 1988) and activity (Capasso et al., 1999) and thus this may be one possible mechanism through which urine flow is reduced in male offspring. Given the correlation we observed between plasma T_4_ and urine flow in male offspring, combined with the lack of changes in *Aqp2* and *Avpr2* mRNA, we suggest that like the studies described above, this change in urine flow is likely driven by a thyroid hormone‐mediated mechanism that currently remains unidentified. Future work will focus on identifying the mechanism through which reduced T_4_ leads to reduced urine flow in our model.

### 4.1 Limitations

We acknowledge that in some analyses, our female data are underpowered. This may have made the detection of differences between female groups more challenging than in male groups and accept that this is a limitation of our study. As we did not examine any parameters indicative of mitochondrial function, like citrate synthase activity or mitochondrial respiration, it is unclear if alterations to Complex II and/or Complex IV protein abundance lead to deficits in mitochondrial function in this model.

## 5 CONCLUSION

This study is the first to demonstrate that maternal selenium deficiency alters protein glycation and the abundance of key proteins involved in mitochondrial function in the kidney in addition to 24 hr urine flow in adult offspring. This study strongly suggests that these findings occur due to impaired glucose handling in female offspring and reduced thyroid hormone concentrations in male offspring. Overall, this study highlights the importance of selenium during development for lifelong health and the prevention of kidney dysfunction.

## CONFLICT OF INTEREST

The authors have nothing to disclose.

## AUTHOR CONTRIBUTIONS

JSMC, AVP, and PH were involved in the initial experiment design, animal experimentation, and tissue collection at Griffith University. JSMC and ESN contributed intellectually to experimental design. ESN performed much of the molecular analysis including urinalysis, Western blotting, and quantification of markers of oxidative stress and mitochondrial function with JSMC at the University of Queensland. ESN and MRA completed qPCR at the University of Queensland. NLK extracted DNA and performed qPCR for determination of mitochondrial content. LAB completed GPX and TXRD protein activity assays and assisted in animal experimentation and tissue collection at Griffith University. ESN performed all statistical analyses, prepared all figures, and wrote the manuscript with JSMC. All authors have approved the final version of the manuscript and agree to be accountable for all aspects of the work. All persons designated as authors qualify for authorship, and all those who qualify for authorship are listed.

## Data Availability

The data that support the findings of this study are available from the corresponding author upon reasonable request.
